# Supplementary material for: Early Use of ECMO for Refractory Kounis Syndrome Concealed by General Anesthesia—A Case Report
Source: Medicina (Kaunas). 2022 Jun 2;58(6):759. doi: 10.3390/medicina58060759 (PMC9227982; doi:10.3390/medicina58060759)
Supplement: Supplementary file 1 [file medicina-58-00759-s001.zip › supplemental_figures and legend(Eng edited).pptx]

## Slide 1
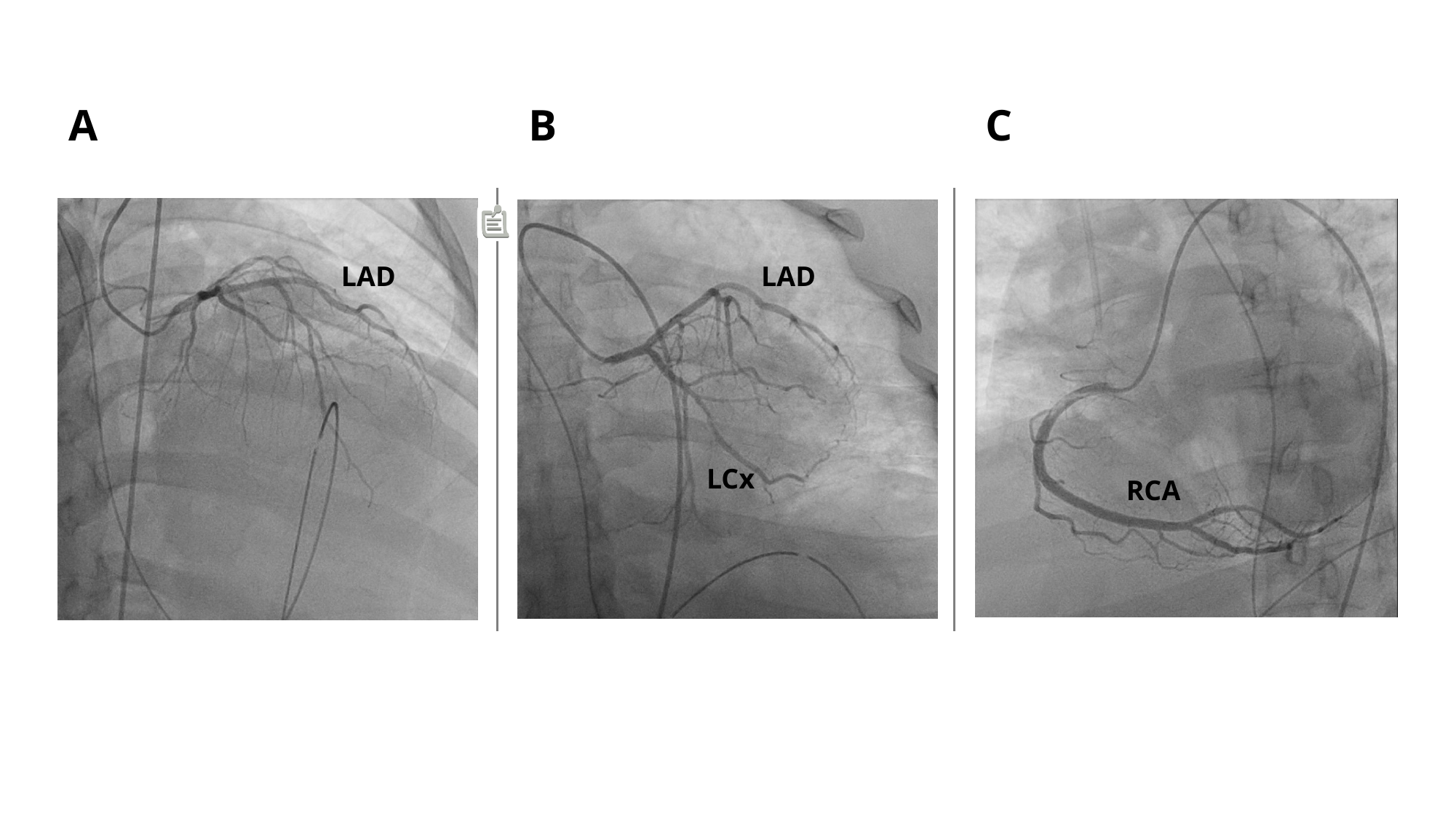

A
B
C
LAD
LAD
LCx
RCA

## Slide 2
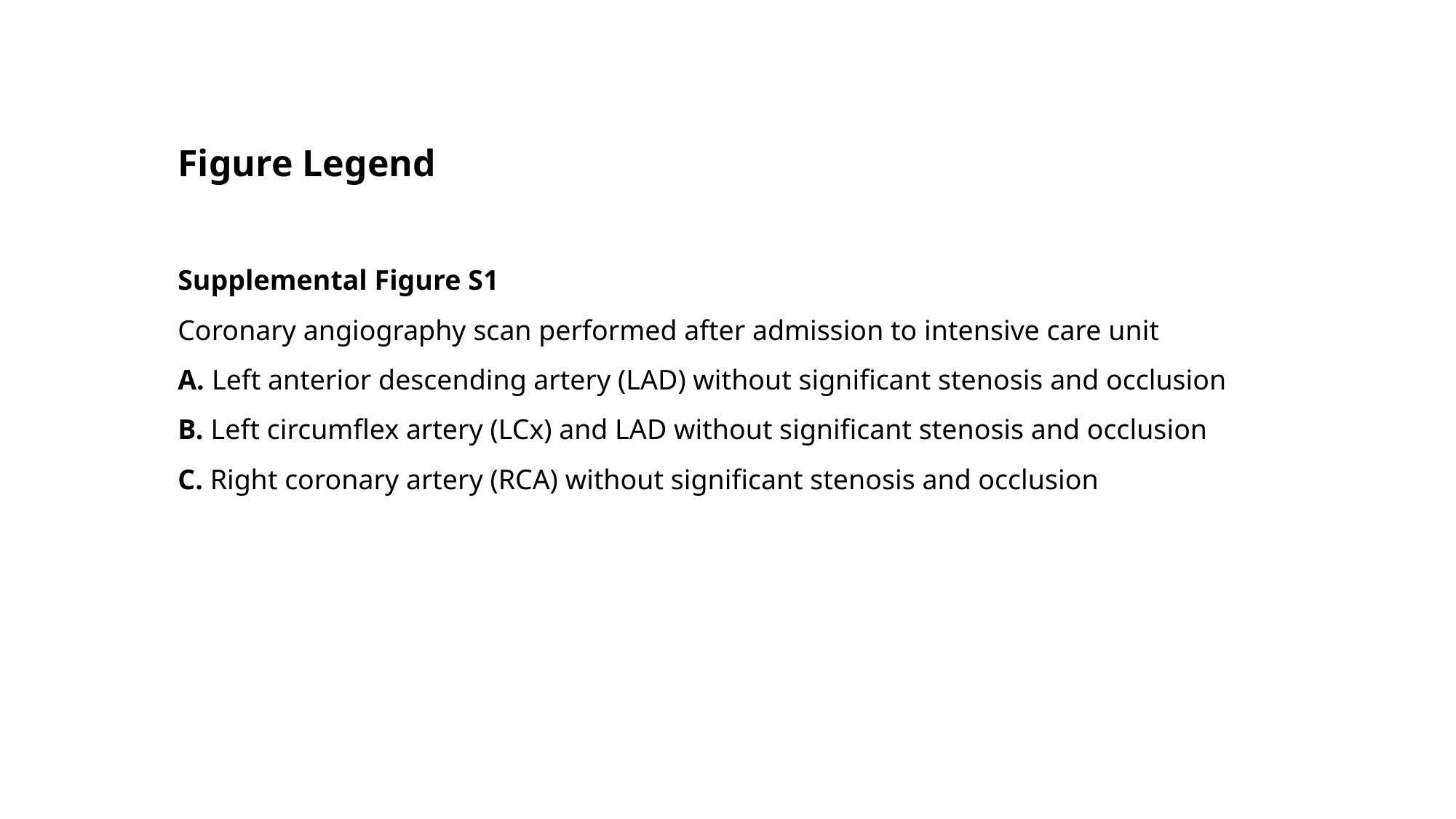

Figure Legend
Supplemental Figure S1
Coronary angiography scan performed after admission to intensive care unit
A. Left anterior descending artery (LAD) without significant stenosis and occlusion
B. Left circumflex artery (LCx) and LAD without significant stenosis and occlusion
C. Right coronary artery (RCA) without significant stenosis and occlusion
